# Single‐cell RNA sequencing elucidated the landscape of breast cancer brain metastases and identified ILF2 as a potential therapeutic target

**DOI:** 10.1111/cpr.13697

**Published:** 2024-06-29

**Authors:** Jindong Xie, Anli Yang, Qianwen Liu, Xinpei Deng, Guangzhao Lv, Xueqi Ou, Shaoquan Zheng, Min‐Yi Situ, Yang Yu, Jie‐Ying Liang, Yutian Zou, Hailin Tang, Zijin Zhao, Fuhua Lin, Wei Liu, Weikai Xiao

**Affiliations:** ^1^ State Key Laboratory of Oncology in South China, Guangdong Provincial Clinical Research Center for Cancer Sun Yat‐sen University Cancer Center Guangzhou China; ^2^ The First Affiliated Hospital Sun Yat‐sen University Guangzhou China; ^3^ Guangdong Provincial Key Laboratory of Malignant Tumor Epigenetics and Gene Regulation, Department of Medical Oncology, Sun Yat‐sen Memorial Hospital Sun Yat‐sen University Guangzhou China; ^4^ Department of Neurosurgery, Xiangya Hospital Central South University Changsha Hunan China; ^5^ Department of Breast, Guangzhou Red Cross Hospital, Medical College Jinan University Guangzhou Guangdong China; ^6^ Department of Breast Cancer, Guangdong Provincial People's Hospital and Guangdong Academy of Medical Sciences Southern Medical University Guangzhou China

## Abstract

Distant metastasis remains the primary cause of morbidity in patients with breast cancer. Hence, the development of more efficacious strategies and the exploration of potential targets for patients with metastatic breast cancer are urgently needed. The data of six patients with breast cancer brain metastases (BCBrM) from two centres were collected, and a comprehensive landscape of the entire tumour ecosystem was generated through the utilisation of single‐cell RNA sequencing. We utilised the Monocle2 and CellChat algorithms to investigate the interrelationships among each subcluster. In addition, multiple signatures were collected to evaluate key components of the subclusters through multi‐omics methodologies. Finally, we elucidated common expression programs of malignant cells, and experiments were conducted in vitro and in vivo to determine the functions of interleukin enhancer‐binding factor 2 (*ILF2*), which is a key gene in the metastasis module, in BCBrM progression. We found that subclusters in each major cell type exhibited diverse characteristics. Besides, our study indicated that *ILF2* was specifically associated with BCBrM, and experimental validations further demonstrated that *ILF2* deficiency hindered BCBrM progression. Our study offers novel perspectives on the heterogeneity of BCBrM and suggests that *ILF2* could serve as a promising biomarker or therapeutic target for BCBrM.

## Graphical abstract

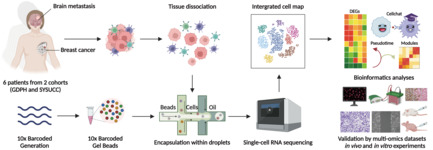

## 1 INTRODUCTION

Breast cancer is the most prevalent form of cancer that affects women worldwide and the second leading cause of cancer‐related death.^1^ Brain metastasis (BrM) is considered the most fatal manifestation of breast cancer and has an approximately 20% one‐year survival rate.^2^ Despite recent advances in systemic treatment, the management of breast cancer brain metastasis (BCBrM) remains a formidable challenge, and available treatments have limited efficacy.^3^, ^4^, ^5^ Hence, increasing our understanding of the fundamental mechanisms associated with BCBrM is imperative.

The investigation of BCBrM progression through bulk transcriptomics is limited by several factors, such as copy number variation and noncancerous cell infiltration, which complicate analyses. Understanding the correlation between malignant cells and the tumour microenvironment (TME) relies on the precise identification and characterisation of distinct cellular states. Furthermore, intratumourigenic heterogeneity plays a crucial role in determining tumour prognosis and progression.^6^, ^7^ In addition, the considerable intratumour heterogeneity among malignant cells introduces significant challenges in accurately discerning genetic diversity through bulk transcriptomics; thus, this a subject of intense controversy. The identification of effective diagnostic markers and therapeutic targets relies on bulk profiling technologies without considering intratumoural heterogeneity; thus, the applicability of these markers and targets to all patients is limited. Single‐cell RNA sequencing (scRNA‐seq) technology provides an accurate way to identify both intrinsic and extrinsic tumour features and is expected to facilitate the resolution of this issue. scRNA‐seq can discern distinct cellular subsets, elucidate clonal diversity, and notably determine the pivotal factors that influence tumour heterogeneity.^8^, ^9^, ^10^ Moreover, the identification of cancer subtypes enables the monitoring of patient’ responses to treatment and the assessment of improvements in clinical outcomes.^11^, ^12^, ^13^, ^14^ Researchers have offered vital insights into BrM biology using scRNA‐seq technology.^15^, ^16^ They identified putative targets for therapeutic modulation, and a rich reference for the BrM research community. Besides, recent advances in other omics methods, such as proteomics and spatial transcriptomics, have also provided novel perspectives on tumour heterogeneity.^17^, ^18^, ^19^ Therefore, it is urgent to apply multi‐omics methods to determine the molecular attributes that are associated with BCBrM progression.

**FIGURE 1:**
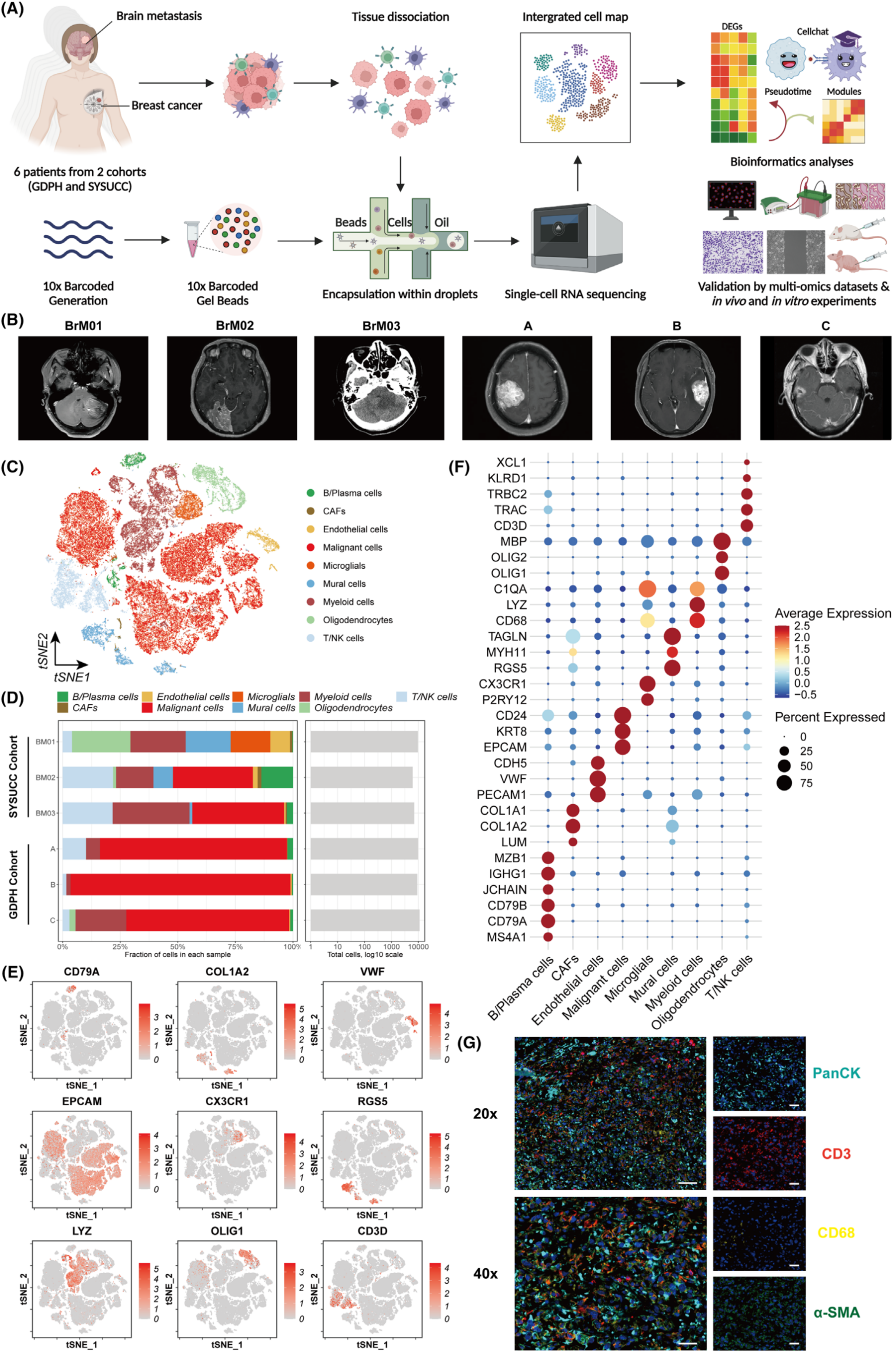
Single‐cell atlas encompassing the cellular composition of BCBrM. (A) Overview of the study design and workflow. Single‐cell suspensions were collected from BCBrM followed by scRNA‐seq on the 10× Genomics platform. (B) The radiomics information (CT and MRI) of each BCBrM patient confirm the occurrence of BrM. (C) t‐SNE plot of single cells profiled in the present study coloured by major cell type. (D) Bar plots showing the relative proportions of each major cell subtype in each sample. (E) Feature plots for the canonical marker genes of B/Plasma cells (*CD79A*), CAFs (*COL1A2*), endothelial cells (*VWF*), malignant cells (*EPCAM*), microglial cells (*CX3CR1*), mural cells (*RGS5*), myeloid cells (*LYZ*), oligodendrocytes (*OLIG1*), and T cells (*CD3D*). (F) Dot plot showing the expression levels of classic marker genes across all major cell types. (G) Representative images of multiplex immunofluorescent staining of BCBrM tissue. The cyan, red, yellow, and green colours indicate positive cells with the expression of PanCK (malignant cells), CD3 (T cells), CD68 (myeloid cells), and α‐SMA (fibroblasts and endothelial cells), respectively, in BCBrM tissue.

Figure 1A shows a schematic representation of the study design and workflow. We conducted thorough analyses of our self‐tested scRNA‐seq datasets that were collected from two centres and include 6 BCBrM patients, and these analyses identified 9 major cell types. We utilised the monocle2 and CellChat algorithms to investigate the interrelationships among the subclusters. In addition, multiple signatures were collected to evaluate key components of the subclusters through multi‐omics methodologies. Finally, we elucidated the common expression programs of malignant cells, and experiments were conducted in vitro and in vivo to determine the functions of interleukin enhancer‐binding factor 2 (*ILF2*), which is a key gene in the metastasis module, in BCBrM progression. Overall, our study offers innovative perspectives on the heterogeneity of BCBrM and reveals the crucial role of *ILF2* in BCBrM progression. This study suggests that *ILF2* might serve as a promising biomarker or therapeutic target for BCBrM.

## 2 METHODS AND MATERIALS

### 2.1 Patients and samples

Six BCBrM samples were obtained from patients who underwent surgery at the Guangdong Provincial People's Hospital and the Sun Yat‐sen University Cancer Center. Table S1 displays the clinical features of all the patients who were included, and Table S2 provides information about the pathology and immunohistochemistry (IHC) of the metastatic tumours. The samples that were used in this study were proven by pathology to be metastatic breast cancer samples. After dissociation of the brain tissue around the tumour, the tumour area was excised for scRNA‐seq analysis. The age range of the participants was 41−55 years, and 48 years was the median age. No patients received chemotherapy or radiation treatment before surgery, except for patient BrM01 (who achieved a pathologic complete response after anti‐HER2 therapy and chemotherapy). All the patients provided informed consent.

### 2.2 Dissociation of tissues and preparation of single‐cell suspensions

Fresh tissues were stored in tissue preservation solution on ice immediately after resection. The tissues were washed three times with HBSS and then digested with 2 mL of tissue dissociation solution at 37°C for 15 min. Red blood cells were removed by incubating the cells at 25°C for 10 min with red blood cell lysis buffer (2 mL). Afterward, centrifugation at 500*g* was carried out 5 min, followed by resuspension of the cells in PBS. Cell viability was evaluated under a microscope using Trypan blue (Sigma).

### 2.3 Library preparation and scRNA‐seq

Single‐cell suspensions were barcoded using the Chromium Single Cell Library, Gel Bead & Multiplex Kit (10× Genomics). The cells were divided into Gel Beads in Emulsion in the ChromiumTM Controller instrument, after which they were lysed and subjected to barcode reverse transcription. The DNA libraries were sequenced on an Illumina HiSeq X instrument with 150‐bp paired‐end reads.

### 2.4 Raw data processing and quality control

The raw reads were processed with Cell Ranger v.3.0.2 to generate gene expression profiles, which were subsequently mapped to GRCh38. FeatureCounts software was used to count genes and UMIs. The cells were filtered according to the default parameters. Analyses of subclusters and cell type annotations were conducted using the ‘Seurat’ R package.^20^

### 2.5 Single‐cell copy number variation (CNV) analysis

The InferCNV method was used to validate CNV and identify malignant cells.^21^ Stromal/immune cells were used as controls. Genes that were expressed in more than 20 cells were sorted according to their position on each chromosome. Based on the residual‐normalized expression values, we normalized relative expression values to 1. We used 1.5 standard deviations as the ceiling to determine the relative expression value. To remove the effects of gene‐specific expression, the relative expression on each chromosome was smoothed using a sliding window.

### 2.6 Pathway enrichment analysis

Each cell subtype was investigated using the ‘clusterProfiler’ R package.^22^ ssGSEA was performed using hallmark pathway sets. Metabolic activity was assessed by the ‘scMetabolism’ algorithm.^23^

### 2.7 Identification of common expression modules

Based on the consensus non‐negative matrix factorization algorithm (cNMF; https://github.com/dylkot/cNMF), transcriptional modules were extracted as meta‐signatures, and their scores were calculated. A meta‐signature was developed, and the samples were hierarchically clustered.

### 2.8 Trajectory and cellular communication analysis

Using the Monocle 2 algorithm, pseudotime trajectory analysis was performed to map subtype differentiation and conversion.^24^ DDRTree was used for dimension‐reduction analysis. Based on known ligand–receptor pairs, we predicted cell‐to‐cell communication between stromal/immune cells and malignant cells.^25^

### 2.9 Data collection and analysis for bulk transcriptome data

Data, including bulk transcriptome data and corresponding clinical information, were downloaded from the GEO database (including GSE22219, GSE209998, GSE43837, GSE100534, GSE148283, GSE173661, GSE42568, and GSE45255).^26^, ^27^ The probes were mapped using the R package ‘AnnoProbe’. The average values of different probes were calculated with the R package ‘limma’.^28^

### 2.10 Cell lines and culture conditions

Breast cancer cell lines (4T1 and MDA‐MB‐231) were obtained from the American Type Culture Collection. A standard protocol was followed to culture all the cell lines, which were maintained at a constant temperature of 37°C without antibiotics. Table S3 lists the sequences of the shRNA and control viruses that were transfected into the cells.

### 2.11 Western blotting analysis

Cells were lysed with RIPA lysis buffer to obtain protein extracts. SDS–PAGE (Beyotime) was used to separate total proteins, and the proteins were transferred to PVDF membranes (Millipore). Antibodies against *ILF2* (14714‐1‐AP) and *GAPDH* (60004‐1‐Ig) were added, and the membranes were incubated at 4°C overnight, followed by incubation with secondary antibodies at room temperature for 1 h. The Immobilon Western Chemiluminescent HRP Substrate (Beyotime) was used to visualise the blots.

### 2.12 Transwell assay

MDA‐MB‐231 and 4T1 cells were digested and reassembled. The experimental cells were added to the upper chambers in fetal bovine serum (FBS)‐free medium, while 20% FBS medium was added to the lower cross‐pore chambers. We analysed all the cells that had migrated after 24 h of fixation with methanol, staining with crystal violet (0.1%), and imaging.

### 2.13 Wound healing assay

MDA‐MB‐231 and 4T1 cells were cultured in six‐well plates and transfected. Following the wound induction procedure, we captured images of the wounds under a microscope at 0, 12, and 24 h, and ImageJ software was used to quantify the wound area and determine cell migration.

### 2.14 Angiogenesis assay

Angiogenesis assays were conducted using endothelial cells (HUVEC and Bend.3 cells). We measured the number of branch points per field after plated cells were exposed to tumour‐conditioned media for a period of 12 h.

### 2.15 Animal experiments

For intracardiac injections, we injected luciferase‐expressing MDA‐MB‐231 cells (in a final volume of 100 mL PBS) into the hearts of 6‐week‐old BALB/c‐nude mice. The BCBrM mouse model was established following a published protocol.^29^ The mice were subcutaneously injected with PBS containing stable *ILF2*‐KD or control 4T1 and MDA‐MB‐231 cells via internal carotid artery injections and were subsequently sacrificed after 2 weeks.

### 2.16 Multiplex immunofluorescent staining

Paraffin‐embedded tissues were utilised for immunofluorescent staining in accordance with established protocols. The paraffin‐embedded sections underwent dewaxing in xylene, rehydration with graded ethanol, blockade of endogenous peroxidase activity, and antigen retrieval at high temperature. Subsequently, the sections were permeabilized in TBST (PBS with 0.5% Triton X‐100) and incubated overnight at 4°C with the primary antibodies. The primary antibodies were administered in a sequential manner, subsequently followed by incubation with the secondary antibody and fluorophore.

### 2.17 IHC staining

Tissue sections were deparaffinized with xylene and rehydrated with ethanol (100%, 95%, 85%, and 75%). Antigen retrieval was performed prior to overnight incubation with the primary antibody at 4°C to block endogenous peroxidase activity. After a 20‐min incubation with an HRP‐conjugated secondary antibody at ambient temperature, staining was performed utilising diaminobenzidine (DAB) substrate (Dako). Following DAB treatment, the sections were stained with haematoxylin.

### 2.18 Statistical analysis

R software was used for statistical analyses, which included Student's *t* test, Pearson correlation test, and Wilcoxon test, and the results are expressed as the means ± standard deviations. For the K–M survival analysis, a log‐rank test was used. *p* < 0.05 was considered to indicate statistical significance.

## 3 RESULTS

### 3.1 Single‐cell atlas encompassing the cellular composition of BCBrM

We analysed BCBrM lesions using scRNA‐seq to determine their cellular composition. Figure 1B shows the radiomics information of each BCBrM patient. After rigorous quality control measures, doublet elimination, and unsupervised clustering, several clusters of cells exhibiting comparable expression patterns were identified (Figures 1C and S1). Classic markers were used to verify that each cluster was a distinct cell population: B/Plasma cells (expressing *CD79A*, *CD79B*, and *MS4A1*), cancer‐associated fibroblasts (CAFs; expressing *LUM*, *COL1A1*, and *COL1A2*), endothelial cells (ECs; expressing *PECAM1*, *VWF*, and *CDH5*), malignant cells (expressing *EPCAM*, *KRT8*, and *CD24*), microglial cells (expressing *P2RY12* and *CX3CR1*), mural cells (expressing *RGS5*, *MYH11*, and *TAGLN*), myeloid cells (expressing *CD68*, *LYZ*, and *C1QA*), oligodendrocytes (expressing *OLIG1*, *OLIG2*, and *MBP*), and T/NK cells (expressing *CD3D*, *TRAC*, *TRBC2*, *KLRD1*, and *XCL1*) (Figures 1E and S1). Dot plots showing cells that expressed cluster‐specific markers are shown according to their expression levels and proportions (Figure 1F). Figures 1D and S1 also show the relative proportions of each major cell subtype in each sample. Interestingly, our findings revealed that oligodendrocytes and malignant cells exhibited the highest number of differentially expressed genes (DEGs), suggesting a notable level of transcriptional variability within these cell types across diverse environments (Figure S1). Additionally, we conducted multiplex immunofluorescent staining to validate the cell subpopulations present in the BCBrM tissue (Figure 1G).

### 3.2 Cell clustering and functional annotation of CAFs in BCBrM

**FIGURE 2:**
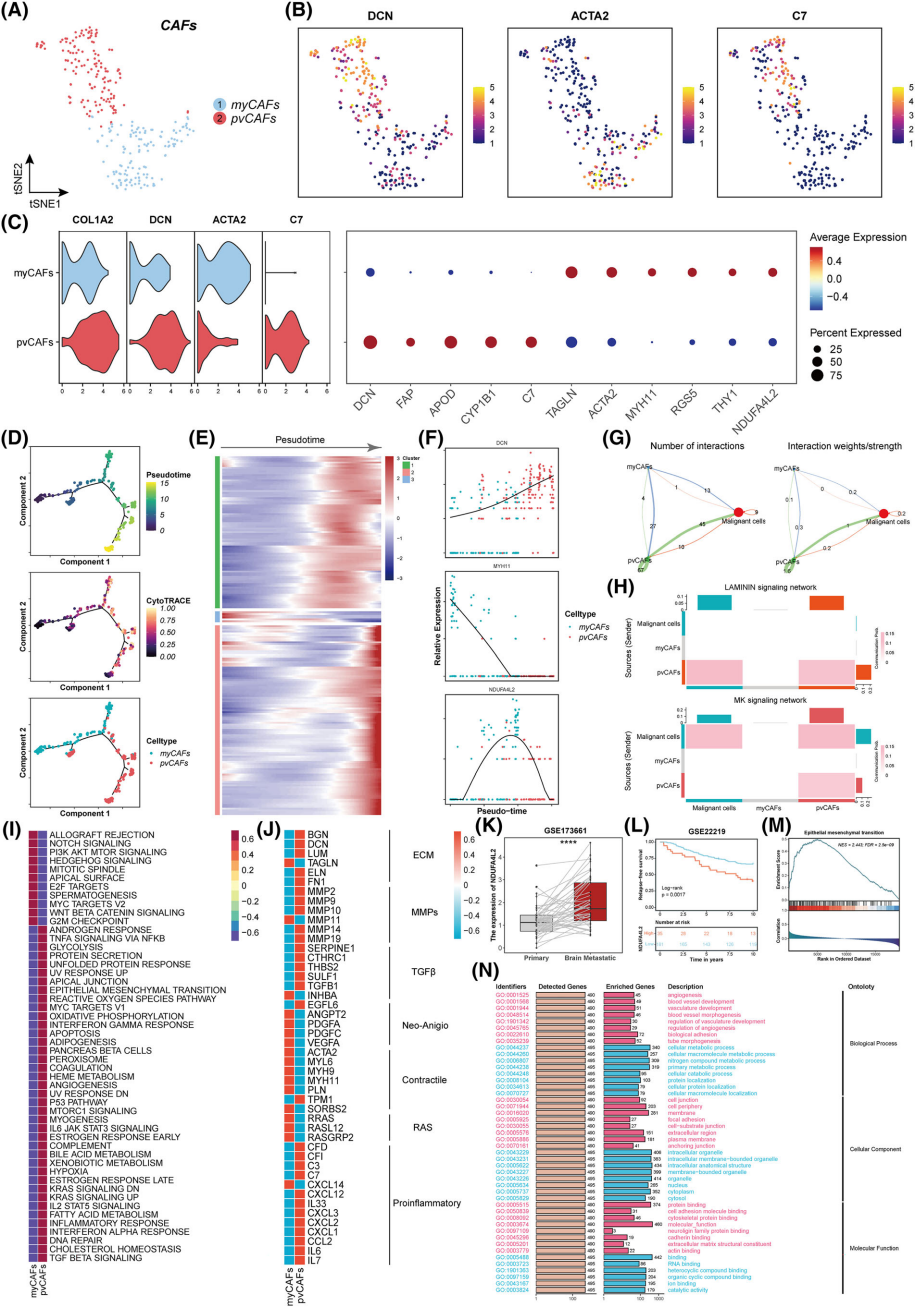
Transcriptional profiling of CAFs in the tumour microenvironment of BCBrM tissues. (A) tSNE plot showing CAFs subtypes, including myCAFs and pvCAFs. myCAFs, myofibroblastic CAFs; pvCAFs, perivascular CAFs. (B) Feature plots showing the expression of selected cluster‐specific genes. (C) Violin plots (left) displaying the representative expression pattern across different subtypes of CAFs. Dot plot (right) showing the expression of the differential gene markers in each subtype. (D) Pseudotime trajectory of CAFs subtypes by Monocle2. Trajectory is coloured by pseudotime (top), CytoTRACE (middle), and cell clusters (bottom). (E) Heatmap showing the scaled expression of differentially expressed genes across pseudotime. (F) The expression of the variable genes involved in the cell state transition. (G) Cellchat analyses showing number of interactions (left) and interaction weights/strength (right) between CAFs subtypes and malignant cells. (H) Heatmap showing the communication probabilities between CAFs subtypes and malignant cells. (I) Heatmap showing the scaled activities of 50 hallmark pathways between CAFs subtypes. (J) Heatmap showing the scaled surface protein genes expression between CAFs subtypes. (K) Box plot showing the expression levels of *NDUFA4L2* between primary and paired BCBrM tissues. **** means *p* < 0.0001. (L) Kaplan–Meier survival analysis of *NDUFA4L2* in GSE22219. (M) Enrichment analyses using gene set enrichment analysis algorithm. NES, normalized enrichment score; FDR, false discovery rate. (N) Enrichment analyses using Gene Ontology database.

CAFs that are present in primary and metastatic tumours demonstrate notable adaptability, plasticity, and resilience, and they actively participate in the progression of cancer through complex interactions with different cell populations within the tumour microenvironment (TME).^30^, ^31^ We reclustered the CAFs and identified two clusters, namely, myofibroblastic CAFs (myCAFs) and perivascular CAFs (pvCAFs), based on the tSNE algorithm (Figure 2A). myCAFs were identified by the expression of classic markers such as *ACTA2* and *TAGLN*, whereas pvCAFs were identified by the expression of *DCN* and *APOD* based on previous research.^32^ We then performed differential gene analysis on the two CAF subtypes and found that myCAFs expressed high levels of *MYH11*, *RGS5*, *THY1*, and *NDUFA4L2*, while pvCAFs expressed high levels of *C7*, *FAP*, and *CYP1B1* (Figures 2B,C, and S2A). In addition, we performed pseudotime trajectory analysis and found that myCAFs and pvCAFs exhibited disparate differential trajectories (Figure 2D), and the heatmap showed that there were different gene expression patterns in different time trajectories (Figure 2E). Furthermore, we examined the changes in gene patterns that were associated with the transitions of each state, as shown in Figure 2F. Then, we conducted cell‐to‐cell communication analyses and found that pvCAFs established more routes of communication with malignant cells than myCAFs (Figure 2G) and that pvCAFs might communicate with malignant cells via LAMININ and MK signalling networks (Figures 2H and S2B,C). Moreover, we assessed the activity levels of 50 hallmark pathways in the two CAF subtypes and found that myCAFs were enriched in biological processes such as the mitotic spindle, notch signalling, and G2M checkpoint pathways, whereas pvCAFs were enriched in the epithelial mesenchymal transition (EMT), glycolysis, and p53 pathways (Figure 2I). Additionally, we compared the expression of several surface proteins and found that pvCAFs were associated with multiple genes related to the extracellular matrix (ECM), matrix metalloproteinases (MMPs), TGF‐β, and proinflammatory factors, while myCAFs were more likely to be correlated with Neo‐Anigio, contractile, and RAS (Figure 2J).

*NDUFA4L2* was highly expressed in myCAFs in our study. We then explored the distribution of *NDUFA4L2* among the scRNA‐seq cohorts of breast cancer patients, and we found that *NDUFA4L2* was mainly expressed in fibroblasts, epithelial cells, and endothelial cells (Figure S2D). Previous studies have confirmed that *NDUFA4L2* can promote tumour progression and is associated with unfavourable survival outcomes.^33^, ^34^ Besides, *NDUFA4L2* can facilitate trastuzumab resistance in HER2‐positive breast cancer.^35^ We then assessed the *NDUFA4L2* expression levels in GSE173661 and found that *NDUFA4L2* was more highly expressed in BCBrM tissues than in paired primary tissues (Figure 2K). The Kaplan–Meier (K‐M) survival analysis results confirmed that *NDUFA4L2* was associated with worse relapse‐free survival (RFS), progression‐free survival (PFS), disease‐free survival (DFS), and overall survival (OS) (Figures 2L and S2E). Furthermore, we performed enrichment analyses and found that *NDUFA4L2* was associated with EMT, angiogenesis, focal adhesion, myogenesis, and so on, which are vital biological processes during BCBrM (Figures 2M,N and S2F,G). Collectively, our analyses revealed the aggregation of distinct CAF subclusters throughout BCBrM.

### 3.3 Immunosuppressive characterization of myeloid cells in BCBrM

**FIGURE 3:**
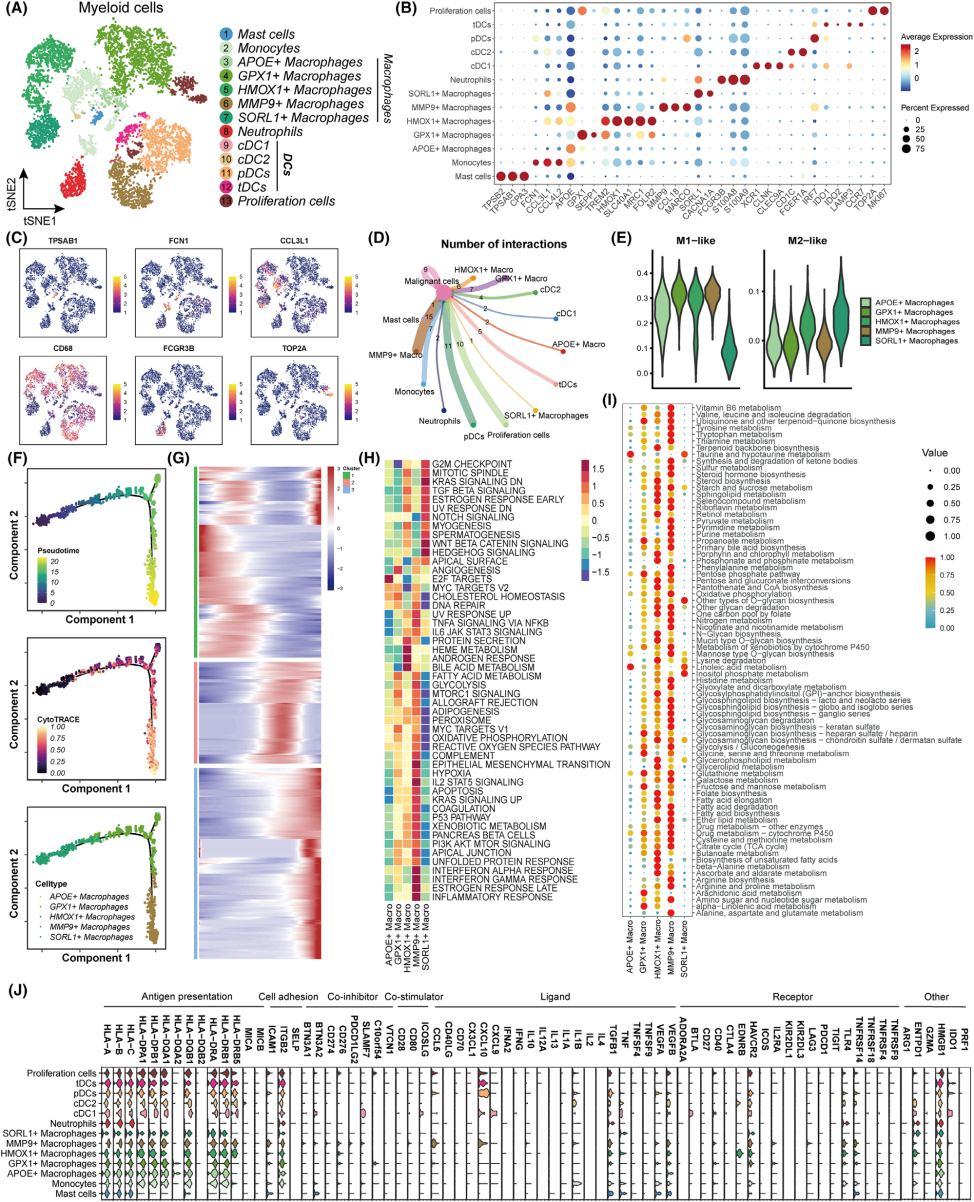
Immunosuppressive characterisation of myeloid cells in BCBrM. (A) tSNE plot showing myeloid cells subtypes, including mast cells, monocytes, macrophages, neutrophils, DCs, and proliferation cells. DCs, dendritic cells. (B) Dot plot showing the expression of the differential gene markers in each subtype. (C) Feature plots showing the normalized expression of highly expressed genes in each myeloid cells subcluster. (D) Cellchat analyses showing number of interactions between myeloid cells subtypes and malignant cells. (E) Violin plots showing the scores of M1‐like and M2‐like signatures among macrophage subtypes. (F) Pseudotime trajectory of macrophage subtypes by Monocle2. Trajectory is coloured by pseudotime (top), CytoTRACE (middle), and cell clusters (bottom). (G) Heatmap showing the scaled expression of differentially expressed genes across pseudotime. (H) Heatmap showing the scaled activities of 50 hallmark pathways between CAFs subtypes. (I) Dot plots showing the metabolic pathways activities among macrophage subtypes. (J) Violin plots showing the scaled immune‐related genes expression among macrophage subtypes.

Subsequently, we analysed the transcriptional attributes of the myeloid cell population in BCBrMs. The myeloid cell population includes mast cells, monocytes, macrophages, neutrophils, dendritic cells (DCs), and proliferating cells, which were further classified into 13 distinct clusters (Figure 3A). Dot plots and feature plots display the markers and proportions of each subtype (Figure 3B,C), and a heatmap shows the most diverse genes within each subtype (Figure S3A). Then, we performed cell‐to‐cell communication analyses and found that each subtype had wide communication networks (Figures 3D and S3B). In addition, we calculated scores for the signatures that were specific to macrophage subtypes, and we found that SORL1+ macrophages were similar to M2‐like macrophages, while *GPX1*+, *MMP9*+, *APOE*+, and *HMOX1*+ macrophages were correlated with M1‐like macrophages (Figures 3E and S3C). Furthermore, Figures 3F and S3D show the results of pseudotime trajectory analysis; we observed that *HMOX1*+ and *SORL1*+ macrophages were in the initial stage of differentiation, while *MMP9*+ and *GPX1*+ macrophages were in the final stages of differentiation. The changes in the expression of genes that are associated with the transitions of each state are also shown in Figure 3G. Additionally, we investigated potentially relevant signalling pathways and biological functions of each subtype using hallmark pathway sets, and we found that *MMP9*+ and *SORL1*+ macrophages exhibited the highest activity of most signalling pathways and biological functions (Figure 3H). Macrophage function and polarization are closely associated with metabolic changes.^36^ By utilising the ‘scMetabolism’ algorithm, we assessed the differences in metabolic pathways among the subtypes. These results indicated that *GPX1*+, *HMOX1*+, and *MMP9*+ macrophages exhibited increased metabolic pathway activity, while *APOE*+ and *SORL1*+ macrophages displayed decreased metabolic pathway activity (Figure 3I). We also assessed the activities of 50 hallmark pathways in other myeloid subclusters, and the results showed that monocytes and neutrophils were enriched in TNF‐α signalling, TGF‐β signalling, and so on, whereas pDCs had activated interferon responses, EMT, and angiogenesis (Figure S3E). It has been documented that myeloid cell are prominent origins of immune checkpoints in tumours.^37^ Therefore, we selected classic immune‐related genes and assessed their expression levels, and the results showed that most of the immune‐related genes were expressed at low levels, except for *ITGB2*, *TGFB1*, *HAVCR2*, *HMGB1*, and genes associated with antigen presentation (Figure 3J). Since the majority of these genes are commonly considered to have immunosuppressive properties, we hypothesised that the immunosuppressive role of myeloid cells limits the efficacy of BCBrM treatment and promotes the progression of BCBrM.

### 3.4 T/NK cells and B/plasma cells were distinguished in BCBrM

**FIGURE 4:**
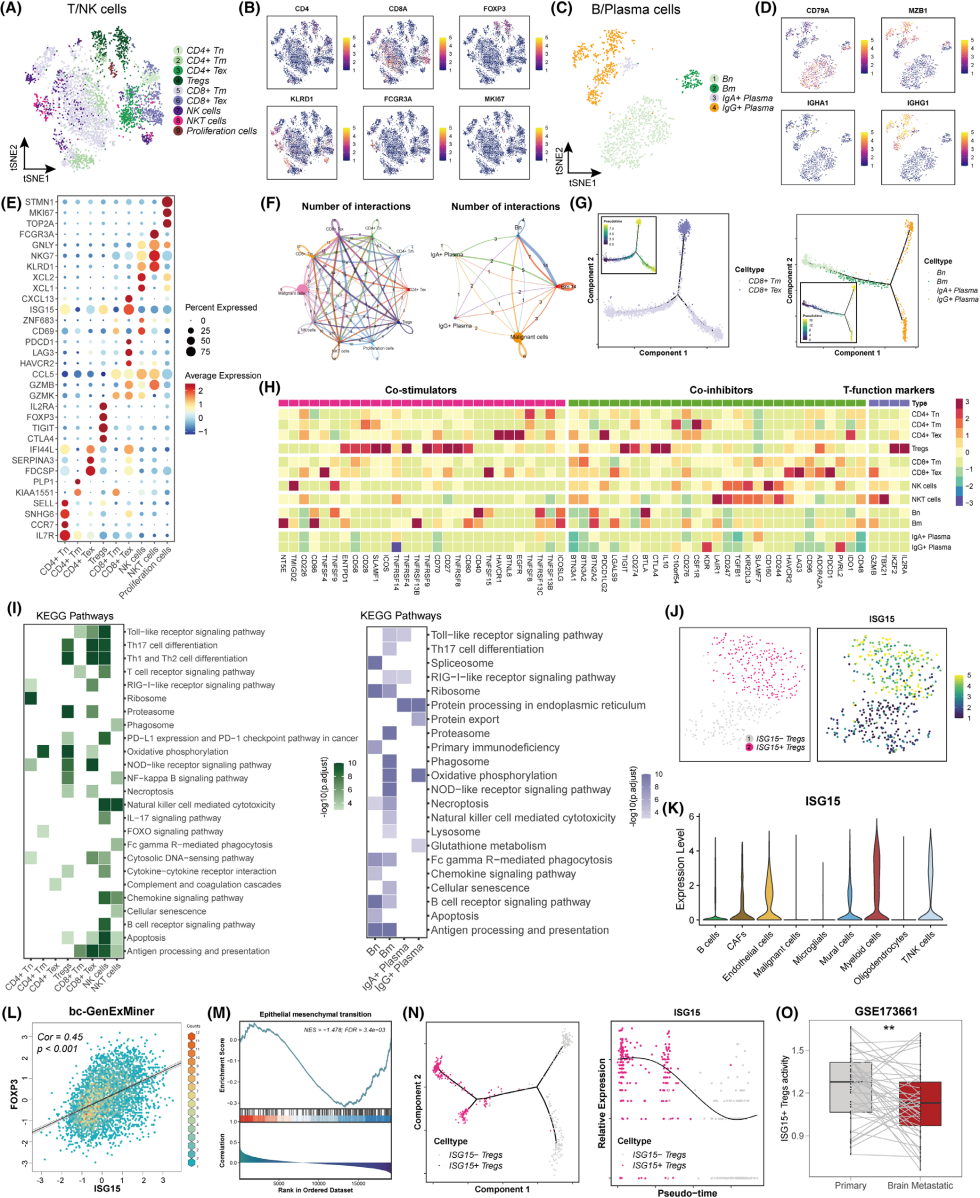
T/NK and B/Plasma cells were distinguished in BCBrM. (A) t‐SNE plot of the T/NK cell landscape. Tn, naïve T cells; Tm, memory T cells; Tex, exhausted T cells. Tregs, regulatory T cells. (B) Feature plots showing the normalized expression of highly expressed genes in each T/NK cell subcluster. (C) t‐SNE plot of the B/Plasma cell landscape. Bn, naïve B cells; Bm, memory B cells. (D) Feature plots showing the normalized expression of highly expressed genes in each B/Plasma cell subcluster. (E) Dot plot showing the expression of the differential gene markers in each T/NK cell subcluster. (F) Cellchat analyses showing number of interactions between T/NK cells (left), B/Plasma cells (right), and malignant cells. (G) Pseudotime trajectory of CD8+ T (left) and B/Plasma (right) cells subclusters by Monocle2. (H) Heatmap showing the scaled expression levels of co‐stimulators, co‐inhibitors, and T‐function markers among T/NK and B/Plasma cells subclusters. (I) Enrichment analyses using KEGG pathways. (J) t‐SNE plot of the Tregs subcluster (left) and feature plots showing the normalized expression of *ISG15* in Tregs subclusters (right). (K) Violin plot showing the expression level of *ISG15*. (L) Correlation analysis between *ISG15* and *FOXP3* using bc‐GenExMiner database. (M) Enrichment analyses using gene set enrichment analysis algorithm. NES, normalized enrichment score; FDR, false discovery rate. (N) The expression of *ISG15* involved in the cell state transition. (O) Box plot showing the differences of *ISG15*+ Tregs activity between primary and paired BCBrM tissues. ** means *p* < 0.01.

To identify high‐resolution subclusters within BrM, we reclustered T/NK cells and B/plasma cells independently. A total of nine subclusters of T/NK cells and four subclusters of B/Plasma cells were identified among the entire patient population (Figure 4A–D). Dot plots and heatmaps provide a summary of the expression of markers in each subcluster (Figures 4E and S4A–C). CD4+ T cell clusters included CD4+ naïve T cells (Tn), CD4+ memory T cells (Tm), CD4+ exhausted T cells (Tex), and regulatory T cells (Tregs); CD8+ T cell clusters consisted of CD8+ Tm and CD8+ Tex cells. In addition, natural killer (NK) cells, along with NKT‐like cells, were also identified. For B/Plasma cells, we obtained naïve B cells (Bn), memory B cells (Bm), IgA+ Plasma cells, and IgG+ Plasma cells. We then performed cell‐to‐cell communication analyses and found that each subtype exhibited wide communication networks (Figures 4F and S4D). In addition, pseudotime trajectory analyses were conducted, and the results showed that each subcluster exhibited different directions (Figures 4G, S4E,F). For example, CD8+ Tm and CD8+ Tex cells had different states; Bn cells were in the initial phase and had the lowest pseudotime score; Bm cells were in the middle phase; and IgA+/IgG+ Plasma cells were during the final phase and exhibited the highest pseudotime score. Moreover, to assess the overall effects of each T/NK and B/Plasma subcluster, we determined the expression levels of immune genes linked to co‐stimulation, co‐inhibition, and specific T function. The results showed that Tregs exhibited both the ‘co‐stimulators’ and ‘co‐inhibitors’ signatures, whereas NK cells, CD8+ T cells, and NKT cells exhibited marked upregulation of ‘co‐inhibitors’ signatures (Figure 4H). Furthermore, we performed enrichment analyses of the T/NK cell and B/Plasma cells subclusters via the KEGG database, and the results confirmed that Tregs, NK cells, NKT cells, and B cells possessed more abundant biological processes (Figure 4I). Moreover, we detected a similar phenomenon via the assessment of hallmark pathway activities (Figure S4G).

Given the vital roles of Tregs in BCBrM that were mentioned above, we further reclustered Tregs and found that Tregs in BCBrM were divided into two subclusters, namely, *ISG15*− Tregs and *ISG15*+ Tregs (Figure 4J). According to our evaluation, *ISG15* was mostly expressed in immune and stromal cells in BCBrM (Figure 4K). The role of *ISG15* in tumour progression is contradictory.^38^, ^39^ We first assessed the expression of *ISG15* and *FOXP3* in breast cancer samples using the bc‐GenExMiner database, and we found a strongly positive correlation between *ISG15* and *FOXP3*, indicating that *ISG15* might be co‐expressing with *FOXP3* (Figure 4L). Since a previous study confirmed that the IFN/EMT signature was associated with the efficacy of immunotherapy in patients,^40^ we then performed enrichment analysis and found that *ISG15* was indeed negatively related to the EMT process (Figures 4M and S4H). Additionally, pseudotime trajectory analyses revealed a transition from *ISG15*+ Tregs to *ISG15*− Tregs in the BCBrM microenvironment (Figure 4N). We also used a single sample gene set enrichment analysis (ssGSEA) algorithm to establish an ‘*ISG15*+ Tregs’ signature and found that the *ISG15*+ Tregs activity scores in BCBrM tissues were significantly higher than that in paired primary breast cancer tissues (Figure 4O). Collectively, T/NK cells and B/Plasma cells were distinguished in BCBrM tissues.

### 3.5 Diversity of mural cells, ECs, and organ‐resident cells in BCBrM

**FIGURE 5:**
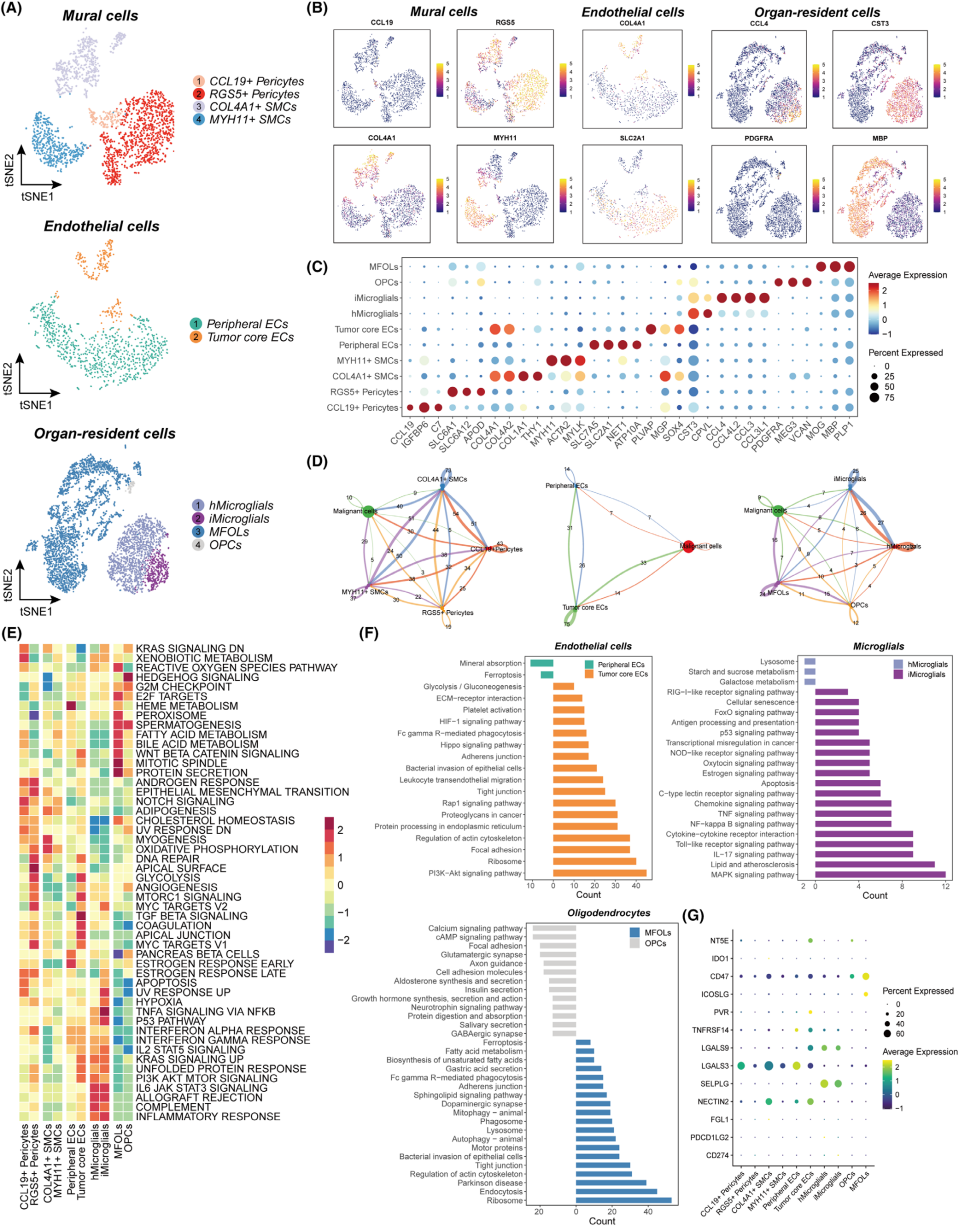
Diversity of mural cells, endothelial cells, and organ‐resident cells in BCBrM. (A) t‐SNE plot of the mural cells (top), endothelial cells (middle), and organ‐resident cells (bottom) landscape. SMCs, smooth muscle cells; ECs, endothelial cells; hMicroglials, homeostatic microglias; iMicroglials, inflammatory microglias; MFOLs, myelin‐forming oligodendrocytes; OPCs, oligodendrocyte progenitor cells. (B) Feature plots showing the normalized expression of highly expressed genes in mural cells (left), endothelial cells (middle), and organ‐resident cells (right) subclusters. (C) Dot plot showing the expression of the differential gene markers in mural cells, endothelial cells, and organ‐resident cells subclusters. (D) Cellchat analyses showing number of interactions between mural cells (left), endothelial cells (middle), organ‐resident cells (right) subclusters, and malignant cells. (E) Heatmap showing the scaled activities of 50 hallmark pathways among mural cells, endothelial cells, and organ‐resident cells subclusters. (F) Enrichment analyses to explore the different biological pathways using KEGG database. (G) Dot plot showing the scaled immune checkpoints expression among mural cells, endothelial cells, and organ‐resident cells subclusters.

Mural cells, ECs, and organ‐resident cells (oligodendrocytes and microglial cells) are essential constituents of the BCBrM microenvironment and actively contribute to the establishment of a distinctive colonized niche and milieu.^41^, ^42^, ^43^, ^44^ For stromal cells, we identified four mural cell subclusters (*CCL19*+ pericytes, *RGS5*+ pericytes, *COL4A1*+ smooth muscle cells [SMCs], and *MYH11*+ SMCs) and two EC subclusters (peripheral ECs and tumour core ECs). For organ‐resident cells, we identified two microglial cell subclusters (homeostatic microglias [hMicroglials] and inflammatory microglias [iMicroglials]) and two oligodendrocytes subclusters (oligodendrocyte progenitor cells [OPCs] and myelin‐forming oligodendrocytes [MFOLs]) (Figure 5A). Peripheral ECs were facilitated by the presence of *SLC2A1*, *SLC7A5*, and *ATP10A*, whereas tumour core ECs were identified by *COL4A1* and *COL4A2*.^45^ For microglial cells, hMicroglials were recognised by *CST3* and *CPVL*, while iMicroglials were identified by inflammatory chemokines such as *CCL4*, *CCL4L2*, and *CCL3*.^46^ For oligodendrocytes, OPCs were characterised by *VCAN*, *MEG3*, and *PDGFRA*, whereas MFOLs were labelled with *MOG*, *MBP*, and *PLP1* ^47^ (Figures 5B,C and S5A–C). Using the CellChat algorithm, we detected distinct interactions among each subcluster (Figure 5D). For example, all mural cell subclusters exhibited broad communication with malignant cells. Among the EC subclusters, tumour core ECs exhibited more communication than did peripheral ECs. Among the oligodendrocyte subclusters, MFOLs demonstrated a higher degree of communication than did OPCs (Figures 5D and S5D). Pseudotime analyses were also performed to verify the transition of cell states (Figure S5E). In addition, we evaluated the activities of hallmark pathways, and the results are shown in Figure 5E. Next, we compared the differentially expressed genes and performed enrichment analyses to explore potentially related biological processes among these subclusters (Figure 5F). We found that tumour core ECs were more highly activated and associated with multiple processes, such as focal adhesion, regulation of actin cytoskeleton, and the PI3K‐AKT signalling pathway. Peripheral ECs were related to mineral absorption and ferroptosis. Among microglial cells, iMicroglials exhibited heightened activation and were linked to cytokine–cytokine receptor interactions and signalling pathways, such as the MAPK, IL‐17, and Toll‐like receptor signalling pathways, etc. iMicroglials were correlated with galactose metabolism, starch and sucrose metabolism, and lysosomes. Among oligodendrocytes, OPCs and MFOLs were both activated. OPCs participated in myelination (calcium and neurotrophin signalling pathway, focal adhesion, axon guidance, etc.), while MFOLs participated in numerous biological processes, such as autophagy, mitophagy, sphingolipid signalling pathway, and ferroptosis. Furthermore, our investigation revealed that *LGALS3* and *NECTIN2* were highly expressed in mural cells; *CD47* and *ICOSLG* were highly expressed in oligodendrocytes; and *LGALS9* and *SELPLG* were highly expressed in microglial cells (Figure 5G). These results uncovered their roles in promoting malignant cell colonization and forming an immunosuppressive microenvironment in BCBrM.

### 3.6 Identification of common modules of malignant cells in BCBrM lesions

**FIGURE 6:**
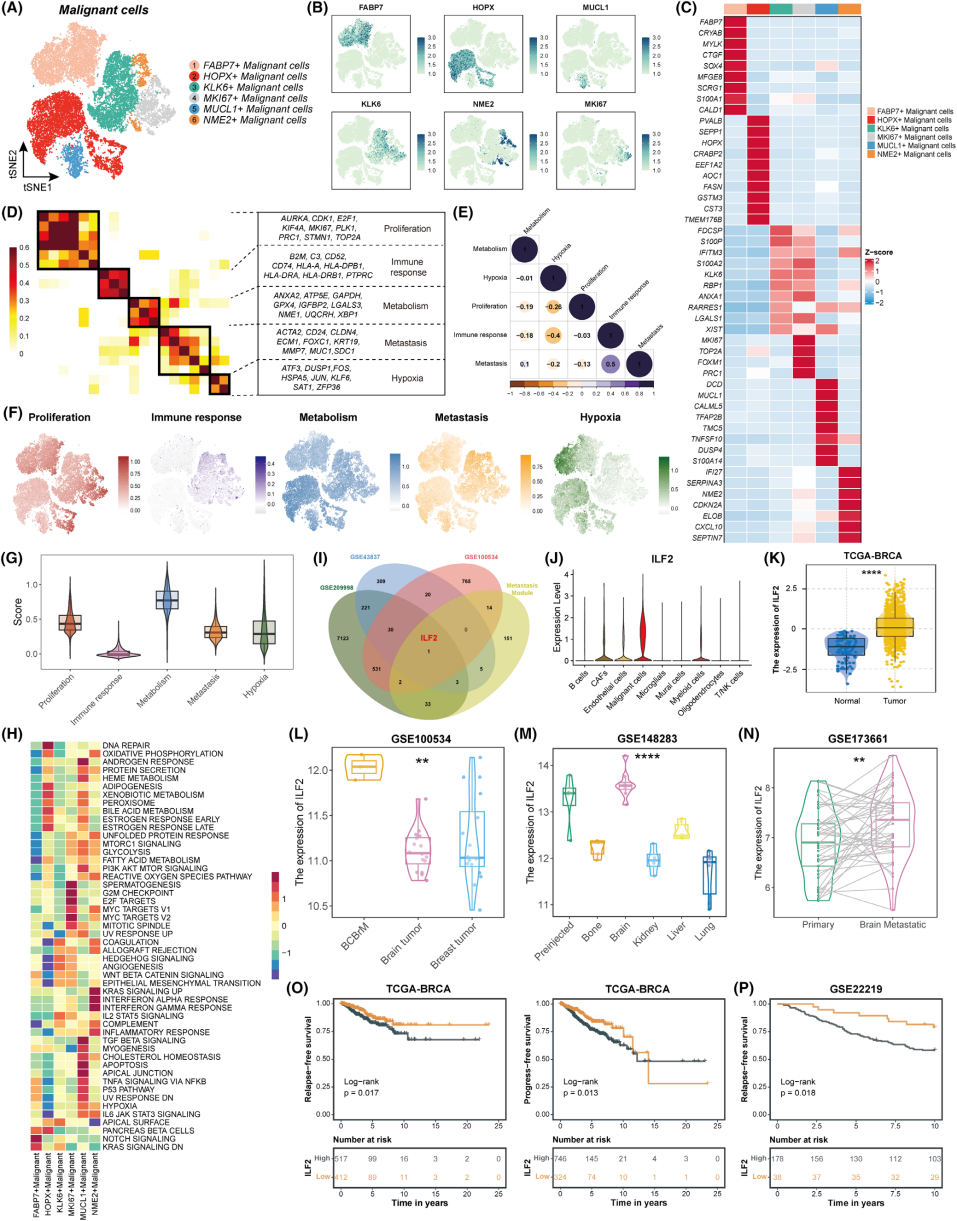
Identification of common modules of malignant cells in BCBrM lesions and found *ILF2* might be a biomarker in BCBrM. (A) t‐SNE plot of the malignant cell landscape. (B) Feature plots showing the normalized expression of highly expressed genes in each malignant cells subcluster. (C) Heatmap of the expression levels of the top differentially expressed genes among subclusters of malignant cells. (D) Heatmap showing five common modules of malignant cells in BCBrM lesion. (E) Heatmap revealing the pairwise interactions among the expression modules. (F) Feature plots showing the distributions of each expression module. (G) Violin plot of the proportions among five expression modules. (H) Heatmap showing the scaled activities of 50 hallmark pathways between malignant cell subclusters. (I) Venn plot showing the intersection gene between BCBrM‐related datasets and the metastasis module. (J) Violin plot showing the expression level of *ILF2*. (K) Box plot showing the expression of *ILF2* between normal and tumour tissues in TCGA‐BRCA dataset. **** means *p* < 0.0001. (L) Violin and box plots showing the expression of *ILF2* among BCBrM, brain tumour in situ, and breast tumour in situ. ** means *p* < 0.01. (M) Violin and box plots showing the expression of *ILF2* among preinjected and metastatic sites. **** means *p* < 0.0001. (N) Violin and box plots showing the expression of *ILF2* between primary and paired BCBrM tissues. ** means *p* < 0.01. (O) Kaplan–Meier survival analyses showing the specific role of *ILF2* in relapse‐free survival and progress‐free survival outcomes in TCGA‐BRCA cohort. (P) Kaplan–Meier survival analysis showing the specific role of *ILF2* in relapse‐free survival outcomes in GSE22219 cohort.

Subsequently, we examined transcriptome patterns and subpopulation clustering to determine the diversity of malignant cells. The InferCNV algorithm was employed to distinguish normal epithelial cells from malignant cells (Figure S6A). Genes with differential expression patterns were annotated into six main subclusters (Figure 6A). We utilised heatmaps and feature plots to present the most differentially expressed genes within each cluster (Figure 6B,C). A meta‐cluster algorithm was developed to identify common modules among BCBrM samples. We compared the most active cancer hallmarks from program cells with those from non‐program cells for each program. Five expression modules were identified, each characterised by distinct biological functions and cell statuses. These modules encompass proliferation, immune response, metabolism, metastasis, and hypoxia (Figure 6D). The proliferation module exhibited elevated gene expression levels of cell cycle‐associated genes such as *AURKA*, *CDK1*, *E2F1*, *KIF4A*, *MKI67*, and *TOP2A*. The immune response module consisted of a series of immune‐related genes (*B2M*, *C3*, *CD74*, and *HLA‐A/DRA*). The metabolism module was characterised by genes related to glutathione homeostasis, mitochondrial metabolism, and cholesterol synthesis (*ANXA2*, *ATP5E*, *GPX4*, *UQCRH*, and *XBP1*). The metastasis module included matrix invasion genes (*MMP7* and *SDC1*) and EMT‐related genes (*CD24* and *KRT19*). The hypoxia module consisted of genes such as *FOS*, *HSPA5*, *JUN*, *KLF6*, and *SAT1*. In addition, pairwise interactions between modules were also verified through correlation analysis (Figure 6E), and the reduced‐dimensional map visually presents the distribution of each expression module (Figure 6F). Furthermore, we conducted a quantitative analysis to determine module scores by assessing the ratio of module cells that corresponded to the level of activity exhibited by these modules in each sample, and the results showed that the ‘immune response’ module generally presented low activity (Figures 6G, S6B,C). Moreover, we assessed the activities of hallmark pathways among these malignant subtypes and observed obvious heterogeneity (Figure 6H). For example, *FABP7*+ malignant cells exhibited high activity in notch signalling pathways; *HOPX*+ malignant cells were enriched in DNA repair and metabolism procedures; *KLK6*+ malignant cells had activated angiogenesis and EMT processes; *MKI67*+ malignant cells were associated with proliferation maintenance; *MUCL1*+ malignant cells were enriched in multiple biological processes, such as the TGF‐β, p53, and TNF‐α signalling pathways; and *NME2*+ malignant cells were related to interferon and inflammatory responses.

### 3.7 *ILF2* is a biomarker of BCBrM

We further conducted bioinformatics analyses to explore genes within the common modules that could be potential biomarkers that could be targeted to inhibit BCBrM. We used the metastasis module and three other GEO datasets (GSE209998, GSE43837, and GSE100534) containing bulk RNA‐seq information from BCBrM samples to identify intersecting genes, and interleukin enhancer binding factor 2 (*ILF2*) was identified as a potential biomarker that could be targeted to inhibit BCBrM (Figure 6I). We initially evaluated the expression of *ILF2* in the scRNA‐seq data and found that *ILF2* was mainly expressed in malignant epithelial cells (Figures 6J and S6D). Additionally, Figure S6E displayed that *ILF2* expression was associated with a greater likelihood of mutations affecting TP53, MUC16, and RYR2. In addition, we found that *ILF2* was upregulated in tumour tissues compared with normal tissues in the TCGA‐BRCA dataset (Figure 6K). Additionally, K–M analyses indicated that *ILF2* was an unfavourable prognostic factor in breast cancer (Figures 6O,P and S6F). Furthermore, using three GEO datasets (GSE100534, GSE148283, and GSE173661), we found that *ILF2* was more highly expressed specifically in BCBrM sites than in primary breast tumours, brain tumours in situ and other metastatic sites, such as the lung, liver, and kidney (Figure 6L–N). These findings were then verified in GSE186344, which is a scRNA‐seq dataset containing the cellular architecture of BrM from different primary sites (Figure S6G). We found that *ILF2* was most highly expressed in metastatic tumour cells (MTCs), except for renal cell carcinoma (RCC) (Figures S6H,I). Taken together, our findings indicated that *ILF2* might be a potential biomarker of BCBrM.

### 3.8 *ILF2* depletion inhibits BCBrM formation in vitro and in vivo

**FIGURE 7:**
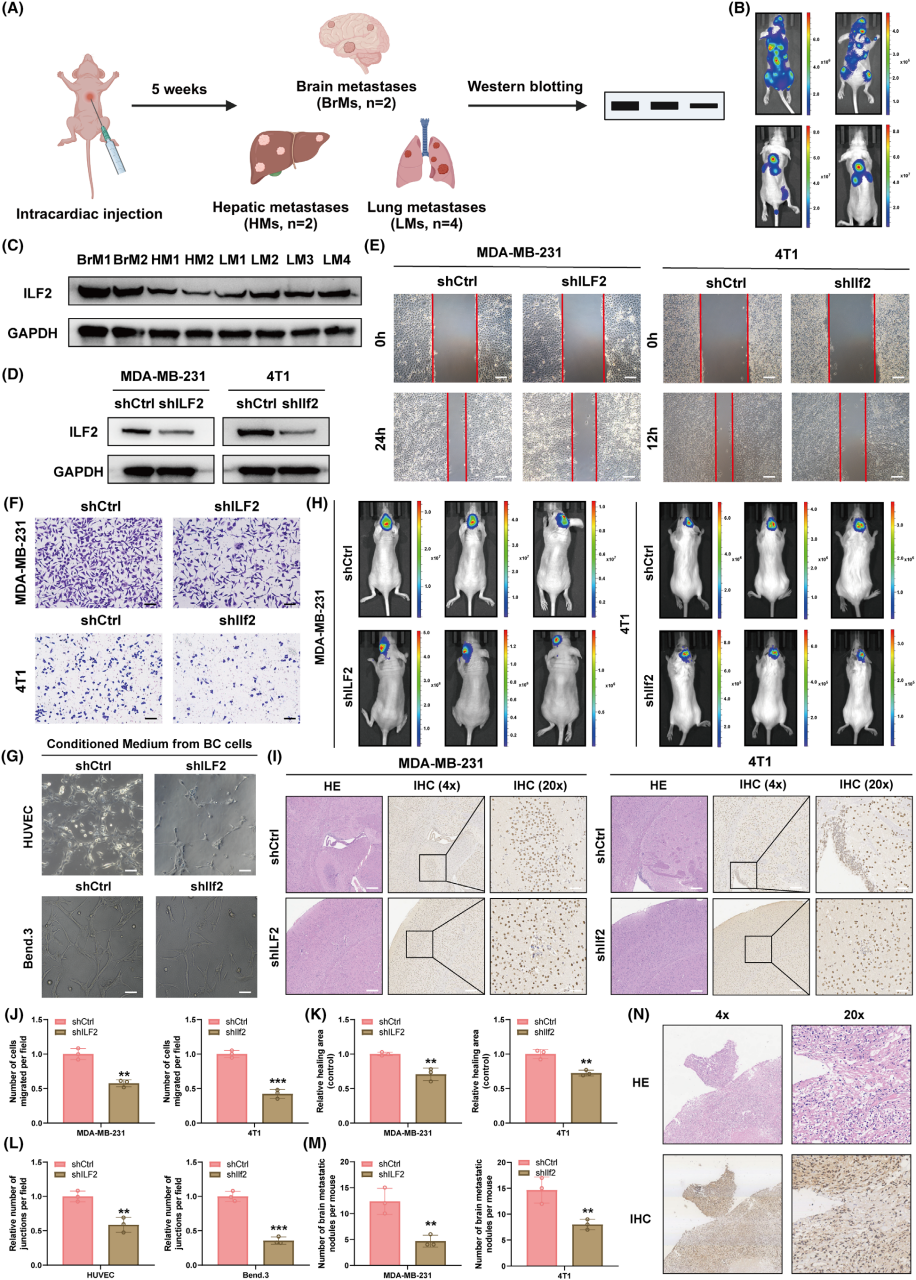
*ILF2* depletion inhibits BCBrM formation in vitro and in vivo. (A) Flow chart indicated that establishment of metastatic mice model and multi‐organ metastases were analysed by western blotting. (B) Bioluminescence imaging showing the multi‐organ metastases of each mouse. (C) The expression *ILF2* in different metastatic sites were determined by western blotting. BrM, brain metastases; HM, hepatic metastases; LM, lung metastases. (D) MDA‐MB‐231 and 4T1 cells were stably transduced with lentivirus encoding short hairpin RNAs targeting *ILF2* (shILF2) or negative control RNA (shCtrl). Then, the cells were assayed for *ILF2* expression. (E) Wound healing assays detected the ability of breast cancer cells to migrate and invade. (F) Transwell assays detected the ability of breast cancer cells to migrate. (G) Angiogenesis assays using HUVEC and Bend.3 cells treated with conditioned media from 4T1 and MDA‐MB‐231 cells treated with shCtrl and shILF2. (H) Bioluminescence imaging showing the BCBrM of each mouse. (I) Haematoxylin Eosin (HE) and immunohistochemistry (IHC) staining of the mice BCBrM tissues. (J, K) Box plots showing differences of number of cells migrated per filed and relative healing area. *** means *p* < 0.001 and ** means *p* < 0.01. (L) Box plots showing differences of number of junctions per field. *** means *p* < 0.001 and ** means *p* < 0.01. (M) Box plots showing differences of number of brain metastatic nodules per mouse. ** means *p* < 0.01. (N) Haematoxylin Eosin (HE) and immunohistochemistry (IHC) staining of the human BCBrM tissue.

Given the multi‐organ metastatic nature of breast cancer and the inherent heterogeneity of cancer cells, we established mouse models of metastasis by injecting MDA‐MB‐231 cells into BALB/c nude mice to determine whether *ILF2* was upregulated in BCBrM in vivo. The flowchart is shown in Figure 7A. Initially, we intracardially injected MDA‐MB‐231 cells into five mice to facilitate the growth of metastatic tumours. After 5 weeks, two mice exhibited hepatic metastases (HMs) and BrMs, whereas four mice displayed lung metastases (LMs) (Figure 7B). The metastases from each mouse were surgically removed, and the extracted proteins were subsequently utilised for western blotting analysis. We observed that *ILF2* was most highly upregulated in BrMs compared with other metastatic sites (Figure 7C). We then explored whether the depletion of *ILF2* could inhibit BCBrM formation. A unique short hairpin RNA (shRNA) was utilised to effectively and stably suppress *ILF2* expression (*ILF2*‐KD) in breast cancer cells (Figure 7D). Subsequently, we observed that *ILF2* depletion significantly inhibited cell migration and invasion (Figure 7E,F,J,K). Given that angiogenesis is a vital component of the metastatic process, we then assessed the potential impact of *ILF2* on the angiogenic properties of BCBrM. Comparing the *ILF2*‐KD conditioned medium with the control medium, we observed a significant reduction in brain endothelial tube formation after exposure to *ILF2*‐KD conditioned medium (Figure 7G,L).

To monitor the in vivo behaviour of tumour cells, we performed bioluminescence imaging (BLI) of luciferase‐tagged *ILF2*‐KD breast cancer cells. Notably, after the cells were injected into mice via the internal carotid artery, the *ILF2*‐KD group exhibited significant inhibition of breast cancer cell growth (Figure 7H). In addition, there was a significant reduction in the size and number of BCBrMs in the *ILF2*‐KD group compared to the control group (Figure 7I,M). Moreover, HE and IHC staining confirmed that *ILF2* possessed highly expression in human BCBrM tissue (Figure 7N). These findings confirmed that *ILF2* was specific for BCBrM and that *ILF2* depletion inhibited BCBrM formation in vitro and in vivo.

## 4 DISCUSSION

There is a stronger immunosuppressive microenvironment within metastatic tumours than within primary tumours due to immune phenotypic shifts, clonal evolution, and organ‐specific niches.^48^, ^49^, ^50^ During colonization, growth, and immune evasion, cancer cells may induce the conversion of normal resident cells into tumour‐promoting cells.^51^ By better understanding metastatic tumours and their associated ecosystem, we could advance the development of more targeted cancer immunotherapy approaches, increasing patient benefits.^52^, ^53^ With recent advances in high‐throughput scRNA‐seq technology and bioinformatics analysis, researchers are now able to comprehensively characterise and explore the complex network of cell‐to‐cell communications within the tumour microenvironment.^54^, ^55^, ^56^ By using scRNA‐seq, primary breast cancer ecosystems can be examined in detail to determine their distinct structure and cellular composition, which holds significant importance in directing the classification of immune phenotypes and revealing the mechanisms underlying the resistance of primary breast cancer to immunotherapy.^57^, ^58^, ^59^ However, limited knowledge exists regarding the mechanisms by which BCBrM cells acclimate to the distinctive microenvironment of the brain.

An extensive analysis of six BCBrM samples led to the identification of a variety of major cell types, namely, B/Plasma cells, CAFs, ECs, malignant cells, microglial cells, mural cells, myeloid cells, oligodendrocytes, and T/NK cells. The molecular characteristics, regulatory mechanisms, temporal changes, and biological roles of each cluster were examined in relation to their involvement in the advancement and immune system evasion of BCBrM. CAFs exhibit loose adherence to arteries, arterioles, venules, and veins in the perivascular space, expressing extracellular matrix proteins while offering structural support.^60^, ^61^ In addition, the remodelling of the extracellular matrix by CAFs and their role in promoting immune evasion within the tumour microenvironment have been extensively documented.^62^, ^63^, ^64^ We found that *NDUFA4L2*+ myCAFs were found in BCBrM and were associated with the EMT process; these cells might accumulate around cancer cells and form a physical barrier that prevents immune cells from entering the core site. We also found that the immunosuppressive role of myeloid cells limited the efficacy of BCBrM treatments and promoted BCBrM progression.^65^ Among T/NK cells, we identified *ISG15*+ Tregs, which might be correlated with BCBrM progression. *ISG15*, which is a ubiquitin‐like protein, was initially identified in 1979 as one of the prominently upregulated *ISG* proteins following stimulation with type I interferon.^66^ A previous study confirmed that *ISG15* could trigger NK cell infiltration and augment MHC‐I expression.^67^ However, the study was performed using immunocompromised mice, which limits the evaluation of the impact of *ISG15* on adaptive immune cells. Our study revealed the transition from *ISG15*+ Tregs to *ISG15*− Tregs in the BCBrM microenvironment, which might lead to immunosuppressive conditions that promote beneficial effects of immunotherapies in patients. Interestingly, different expression levels of immune checkpoints were observed among mural cells, ECs, and organ‐resident cells. For malignant cells, we distinguished five common modules with diverse biological functions and cell statuses, including proliferation, immune response, metabolism, metastasis, and hypoxia. Among these hub genes, *ILF2* was identified and validated as an effective target for inhibiting BCBrM progression. *ILF2* serves as a crucial transcription factor that is necessary for the T cell‐specific expression of interleukin 2. *ILF2* functions as a regulatory cofactor that engages with various partners that are involved in the regulation of gene expression, operating at both the transcriptional and posttranscriptional levels. Previous studies have highlighted its oncogenic role in numerous malignant diseases, such as non‐small cell lung cancer, multiple myeloma, oesophageal cancer, and pancreatic ductal adenocarcinoma.^68^, ^69^, ^70^, ^71^ However, its role in breast cancer has received little attention. Our study confirmed that *ILF2* was specifically expressed in BCBrM and that *ILF2* depletion inhibited BCBrM formation in vitro and in vivo; thus, *ILF2* might serve as a promising biomarker or therapeutic target for BCBrM.

Notably, limitations still exist in this study and our results need further investigation. First, large‐scale (both clinical and animal) samples should be used to verify our findings. Second, further validations using novel omics methods (metabolomics, proteomics, spatial transcriptomics, etc.) and experiments are necessary to verify our findings. Third, since metastasis is a complex process contains multiple steps (including in situ tumour growth, angiogenesis, EMT, invasion, infiltration, circulation survival, extravasation, dormancy, and metastatic tumour growth),^72^, ^73^ it is imperative to determine the complex underlying mechanisms by which *ILF2* affects BCBrM progression. Moreover, how to achieve clinical transformation by targeting *ILF2* still needs to be considered.

## 5 CONCLUSION

Taken together, our findings provide novel insights into the heterogeneity of BCBrM, and reveal the crucial role of *ILF2* in BCBrM progression. Besides, we found that *ILF2* might serve as a promising biomarker or therapeutic target for BCBrM.

## AUTHOR CONTRIBUTIONS

*Research design*: Weikai Xiao, Wei Liu, Fuhua Lin, Zijin Zhao, and Hailin Tang. *Data collection*: Jindong Xie, Anli Yang, Qianwen Liu, and Xinpei Deng. *Data analysis*: Jindong Xie, Anli Yang, and Qianwen Liu. *Manuscript preparation*: Jindong Xie, Anli Yang, Qianwen Liu, Xinpei Deng, Guangzhao Lv, Xueqi Ou, Shaoquan Zheng, Min‐Yi Situ, Yang Yu, Jie‐Ying Liang, Yutian Zou. *Manuscript editing*: Weikai Xiao, Wei Liu, Fuhua Lin, Zijin Zhao, and Hailin Tang. All authors contributed to the article and approved the submitted version.

## FUNDING INFORMATION

This study was supported by funding from the National Natural Science Foundation of China (82003066, Weikai Xiao; 82203127, Anli Yang; 82104225, Zijin Zhao), Guangzhou Science and Technology Plan Project (2024A03J0674, Wei Liu), the Research‐oriented Hospital Program of Guangzhou (RHPG05, Wei Liu), Basic and Applied Research Fund of Guangdong Province (2023A1515111052, Wei Liu), and the Postdoctoral Fellowship Program of CPSF (GZB20230903, Shaoquan Zheng).

## CONFLICT OF INTEREST STATEMENT

The authors declare no conflicts of interest.

## PATIENT CONSENT STATEMENT

Ethics committee of Sun Yat‐sen University Cancer Center and Guangdong Provincial People's Hospital approved this study.

## Supporting information


**Data S1.** Supporting information.

**Data S2.** Supporting information.

## Data Availability

The data that support the findings of this study are available on request from the corresponding author.
