# Supplementary material for: Single‐cell RNA sequencing elucidated the landscape of breast cancer brain metastases and identified ILF2 as a potential therapeutic target
Source: Cell Prolif. 2024 Jun 29;57(11):e13697. doi: 10.1111/cpr.13697 (PMC11533045; doi:10.1111/cpr.13697)

Supplementary Figure legends

Figure S1. Single-cell atlas encompassing the cellular composition of BCBrM. (A) t-SNE plot of single cells profiled in the present study colored by cohort (left) and sample (right). **(B)** Feature plots for the canonical marker genes of B/Plasma cells (CD79B), CAFs (COL1A1), endothelial cells (CDH5), malignant cells (KRT8), microglial cells (P2RY12), mural cells (TAGLN), myeloid cells (CD68), oligodendrocytes (OLIG2), and T cells (TRAC). **(C)** Heatmap of the expression levels of the top differentially expressed genes among subclusters of major cell types. **(D)** Bar plots showing the relative proportions of each major cell subtype in each cohort. **(E)** t-SNE plot showing the distribution of major cell types (left) and the number of differentially expressed genes (DEGs) in each cell type (right).

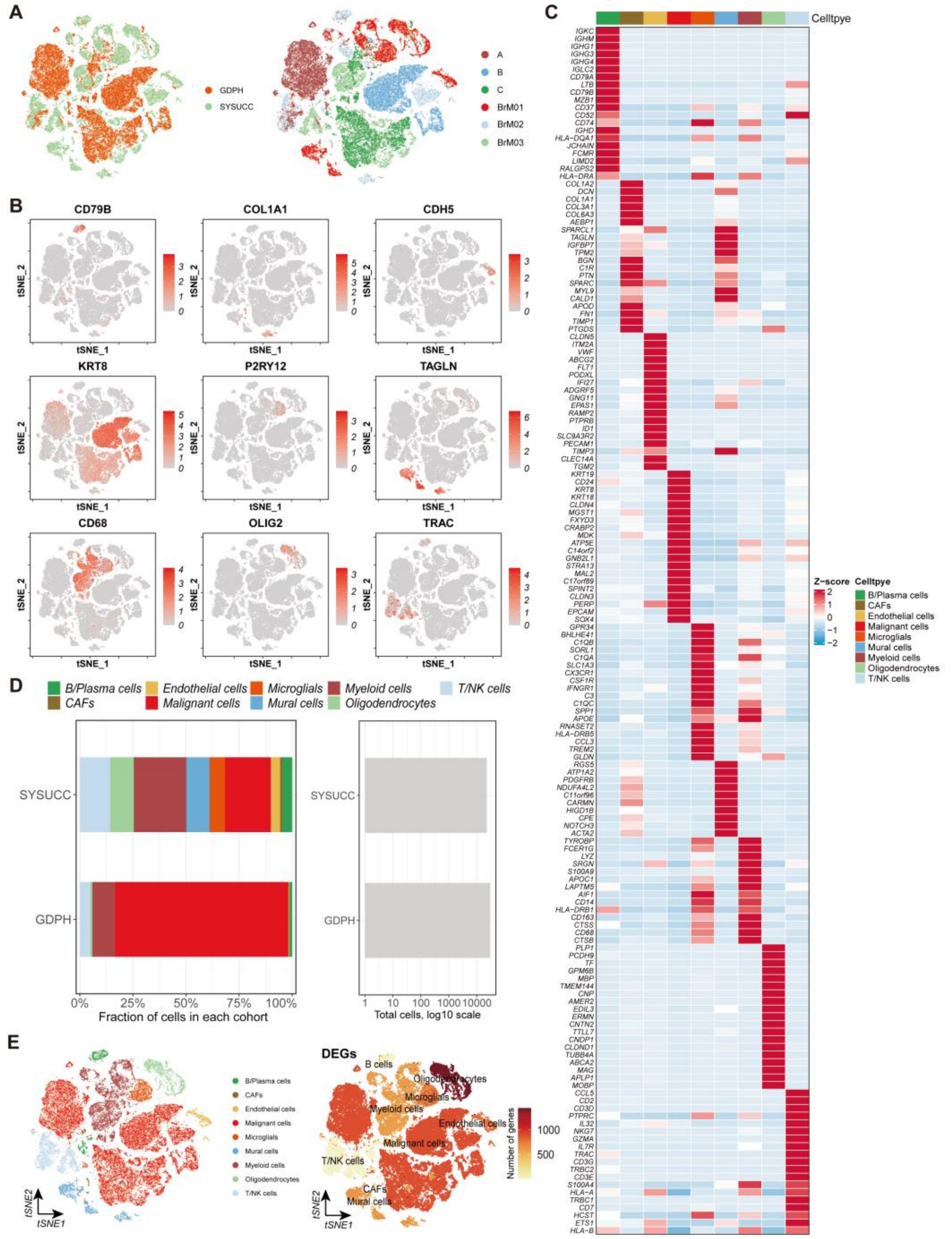

Figure S2. Transcriptional profiling of CAFs in the tumor microenvironment of BCBrM tissues. (A) Heatmap of the expression levels of the top differentially expressed genes among subclusters of CAFs. **(B)** Cellchat analaysis showing outgoing (left) and incoming (right) signaling patterns between CAFs subtypes and malignant cells. **(C)** Violin plots showing the expression levels of related genes in LAMININ (left) and MK (right) signaling patterns. **(D)** Heatmap of the expression levels of NDUFA4L2 among scRNA-seq datasets using TISCH database. **(E)** Kaplan-Meier survival analysis of NDUFA4L2 in GSE42568, GSE45255, and TCGA-BRCA datasets. **(F)** Enrichment analyses using KEGG database. **(G)** Enrichment analyses using gene set enrichment analysis algorithm. NES, normalized enrichment score; FDR, false discovery rate.

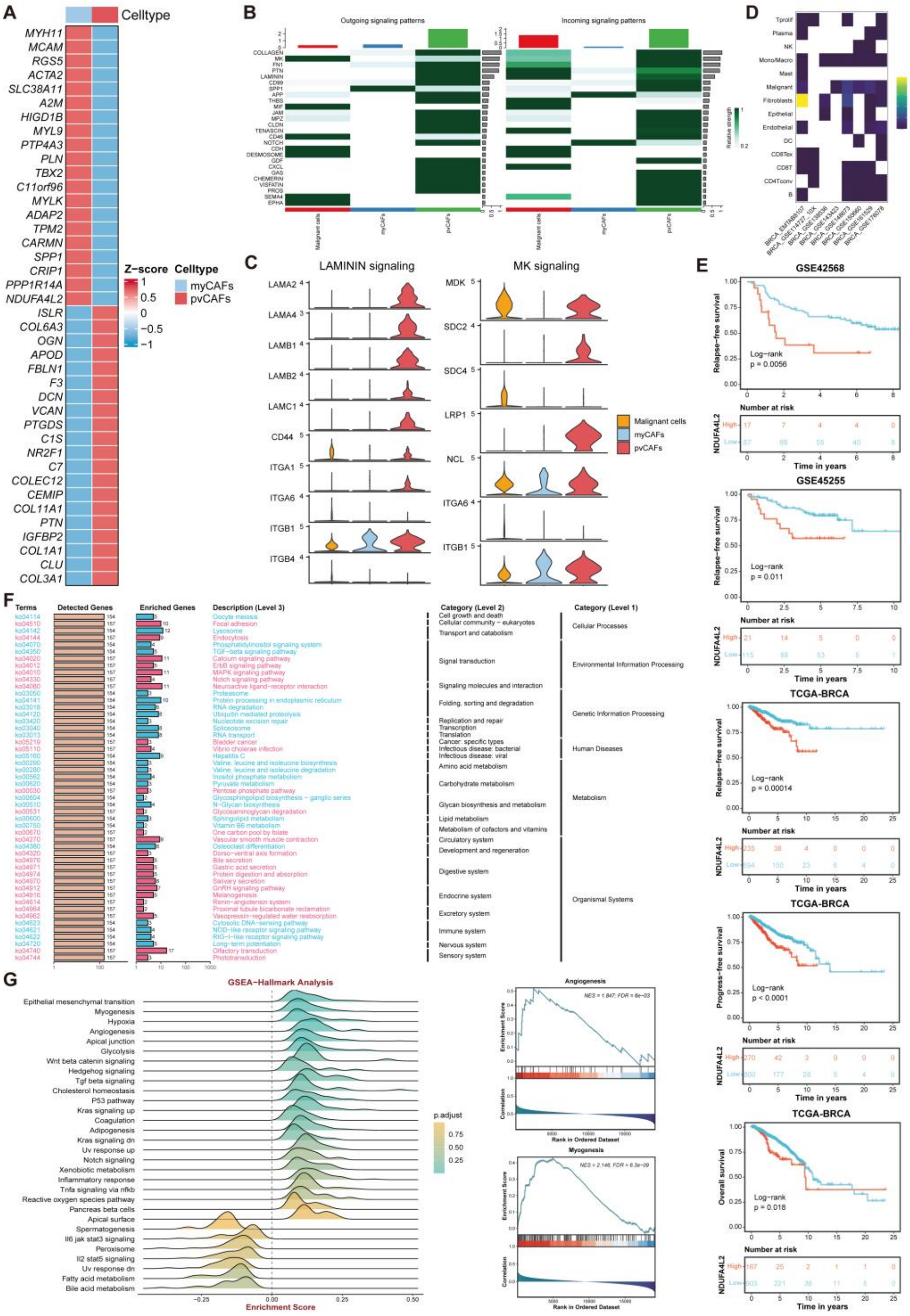

Figure S3. Immunosuppressive characterization of myeloid cells in BCBrM. (A) Heatmap of the expression levels of the top differentially expressed genes among subclusters of myeloid cells. **(B)** Cellchat analaysis showing outgoing (left) and incoming (right) signaling patterns between myeloid cells subtypes and malignant cells. **(C)** tSNE plot showing macrophages subtypes (left), and feature plots showing the scores of M1-like (middle) and M2-like (right) signature among macrophages subtypes. **(D)** The expression of the variable genes involved in the cell state transition. **(E)** Heatmap showing the scaled activities of 50 hallmark pathways between other myeloids subtypes.

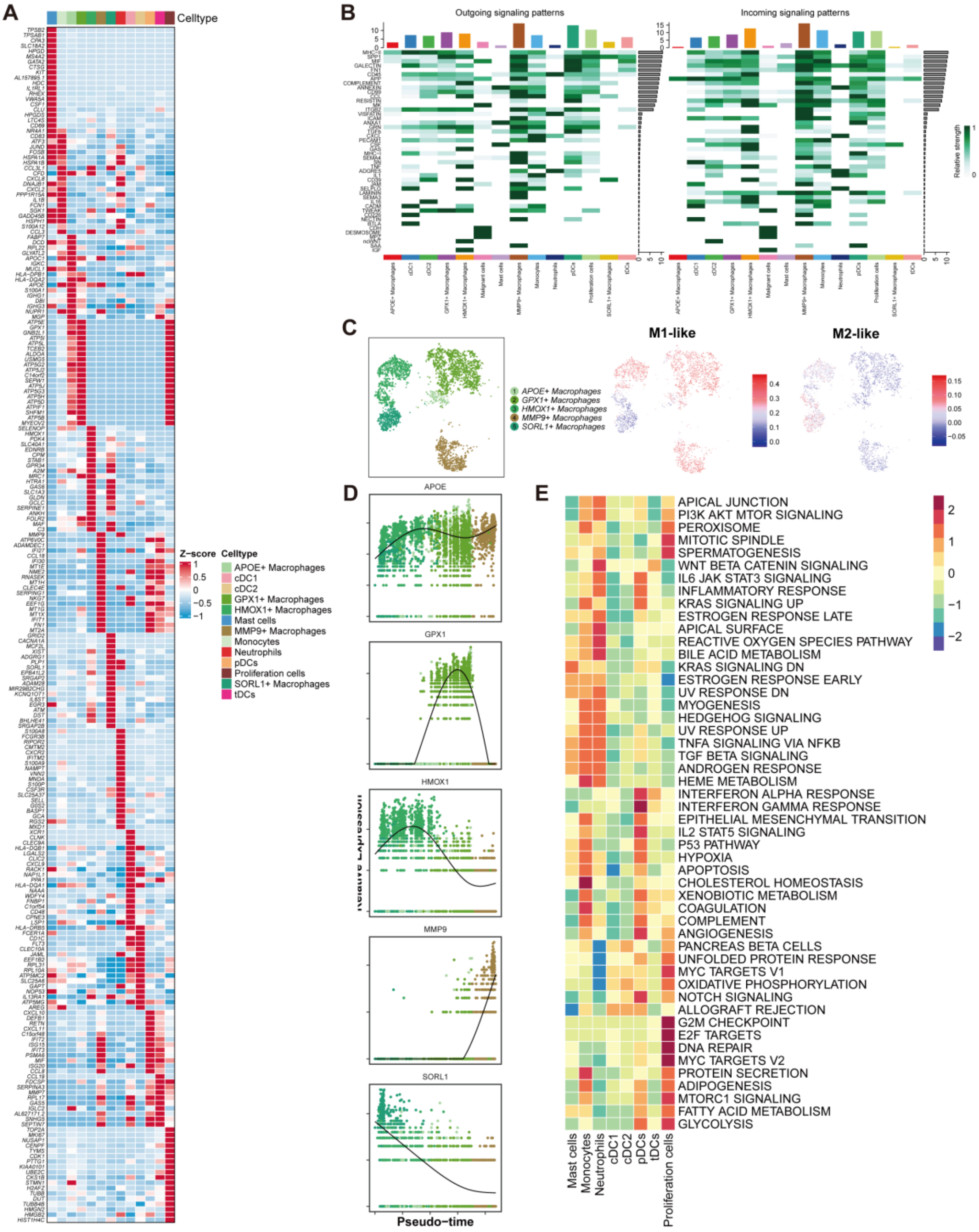

Figure S4. T/NK and B/Plasma cells were distinguished in BCBrM. (A) Dot plot showing the expression of the differential gene markers in each B/Plasma subtype. **(B-C)** Heatmap of the expression levels of the top differentially expressed genes among subclusters of T/NK and B/Plasma subtypes. **(D)** Cellchat analaysis showing outgoing (left) and incoming (right) signaling patterns between T/NK subtypes (top), B/Plasma subtypes (bottom), and malignant cells. **(E)** Pseudotime trajectory of CD4+ T cells subclusters by Monocle2. **(F)** The expression of the variable genes involved in the cell state transition. **(G)** Heatmap showing the scaled activities of 50 hallmark pathways among T/NK subtypes (left), B/Plasma subtypes (right). **(H)** Enrichment analyses using gene set enrichment analysis algorithm. NES, normalized enrichment score; FDR, false discovery rate.

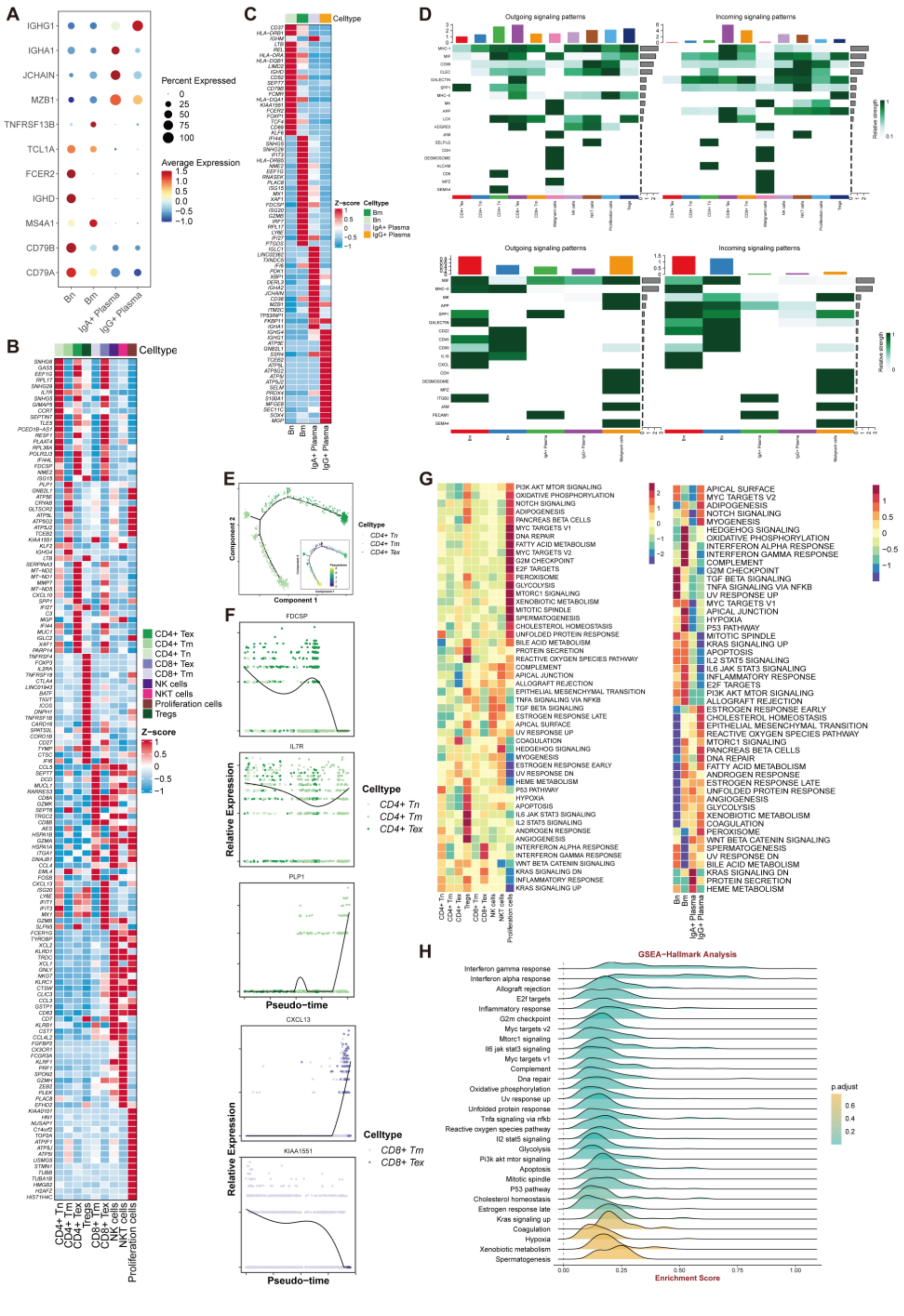

Figure S5. Diversity of mural cells, endothelial cells, and organ-resident cells in BCBrM. (A-C) Heatmap of the expression levels of the top differentially expressed genes among subclusters of mural cells, endothelial cells, and organ-resident cells. **(D)** Cellchat analaysis showing outgoing (left) and incoming (right) signaling patterns between mural cells subtypes (top), endothelial cells subtypes (middle), and organ-resident cells subtypes (bottom) and malignant cells. **(E)** Pseudotime trajectory of mural cells, endothelial cells, and organ-resident cells subtypes by Monocle2. Trajectory is colored by pseudotime (top), CytoTRACE (middle), and cell clusters (bottom).

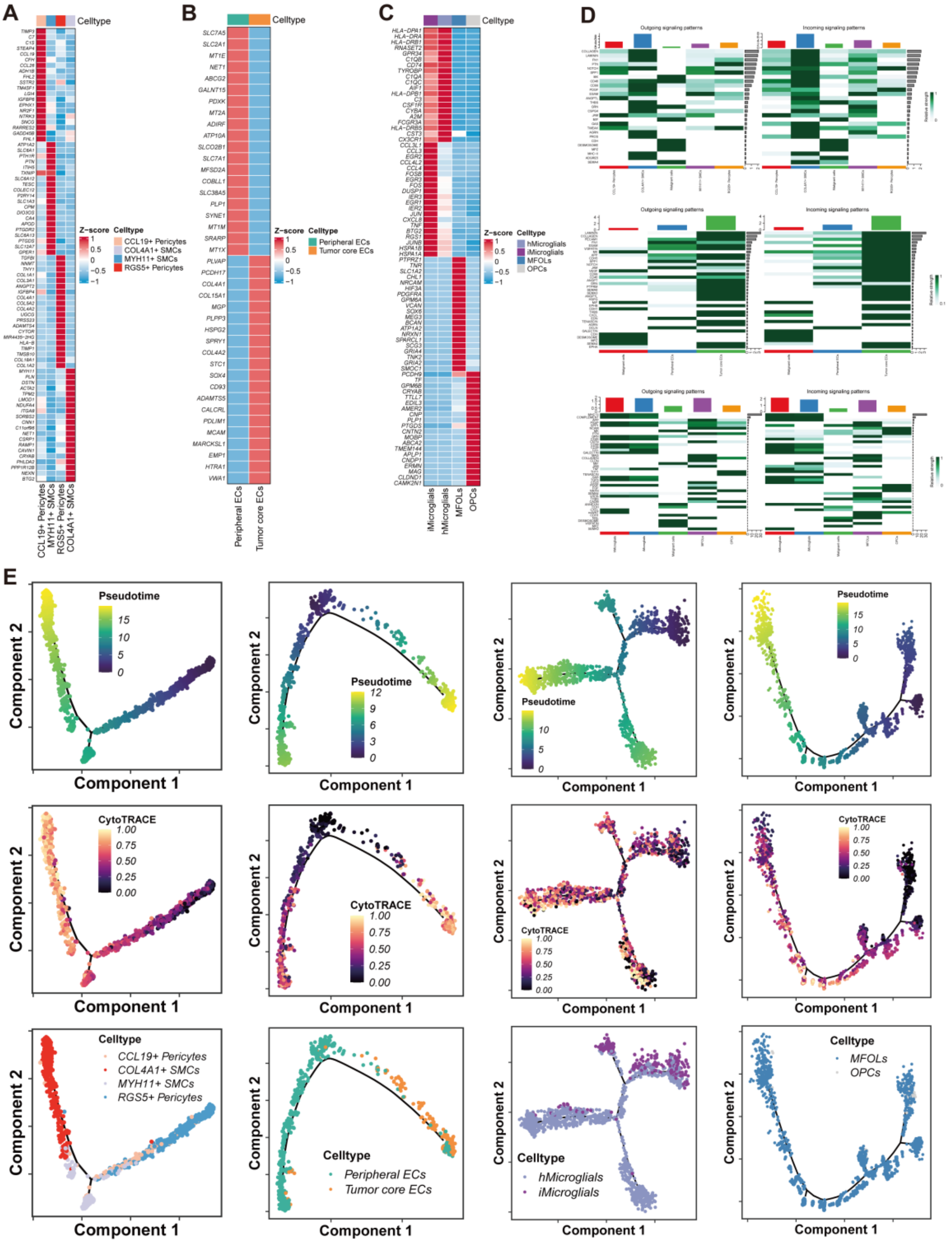

Figure S6. Identification of common modules of malignant cells in BCBrM lesions and found ILF2 might be a biomarker in BCBrM. (A) Identification of malignant cells using InferCNV method. **(B-C)** Violin plots and heatmap showing the general activities of each module among malignant cells subclusters. **(D)** Heatmap of the expression levels of ILF2 among scRNA-seq datasets using TISCH database. **(E)** Waterfall plot showing the mutation differences based on the expression of ILF2 in TCGA-BRCA dataset. **(F)** Kaplan-Meier survival analysis of ILF2 in TCGA-BRCA, GSE42568, and GSE45255 datasets. **(G)** t-SNE plot of the major cell types (left) and primary sites (right) in GSE186344. **(H)** Feature plots showing the normalized expression of ILF2 in GSE186344. **(I)** Violin plot showing the expression level of ILF2 in each cell type which was split by primary sites.

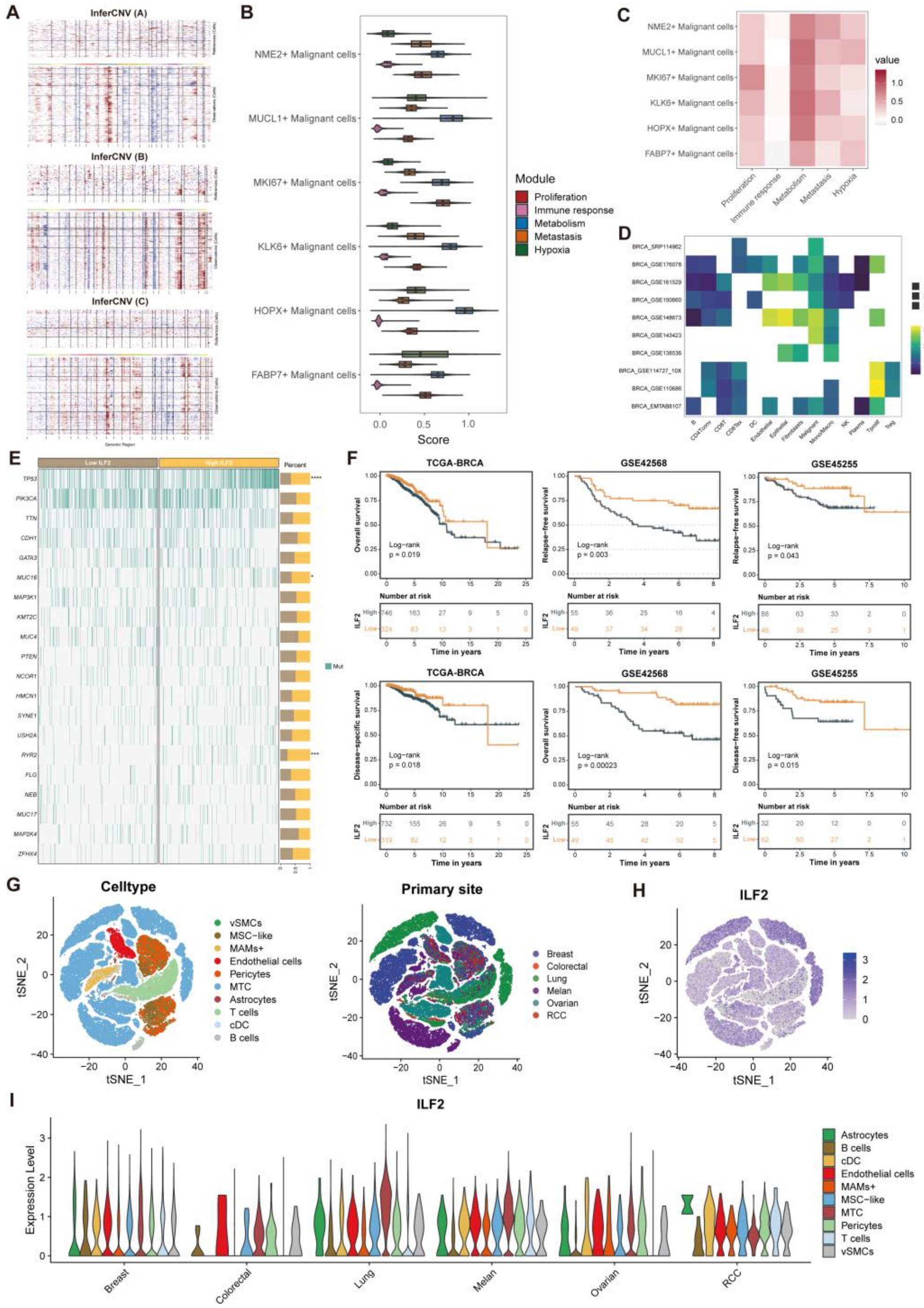

Supplement: Supplementary file 1 — Data S1. Supporting information. [file CPR-57-e13697-s001.pdf]
