# Supplementary material for: Single‐cell RNA sequencing elucidated the landscape of breast cancer brain metastases and identified ILF2 as a potential therapeutic target
Source: Cell Prolif. 2024 Jun 29;57(11):e13697. doi: 10.1111/cpr.13697 (PMC11533045; doi:10.1111/cpr.13697)
Supplement: Supplementary file 2 — Data S2. Supporting information. [file CPR-57-e13697-s002.pdf]

Table S1. Clinical information for metastatic breast cancer patients analyzed by scRNA-seq in this study.

Case ID	Gender	Age	Cohort	Metastatic site	Metastasis pattern	Location	Pre-treatment of metastatic disease
BrM01	Female	48	SYSUCC	Brain	Heterochronous	Left cerebellar hemisphere	Trastuzumab, Pertuzumab, nab-paclitaxel
BrM02	Female	54	SYSUCC	Brain	Heterochronous	Right occipital lobe	/
BrM03	Female	41	SYSUCC	Brain	Heterochronous	Left cerebellar hemisphere	/
A	Female	55	GDPH	Brain	Heterochronous	Left temporal lobe	/
B	Female	54	GDPH	Brain	Heterochronous	Bilateral temporal lobes	/
C	Female	48	GDPH	Brain	Heterochronous	Right temporal lobe	/

Supplementary Table S2. Pathological and immunohistochemical information for BCBrM patients analyzed by scRNA-seq in this study.

Case ID	ER	PR	HER2 (IHC)	Ki67
BrM01	/	/	/	/
BrM02	0	0	3+	70%+
BrM03	0	0	0	60%+
A	50%+	0	2+	20%+
B	0	0	0	60%+
C	0	0	0	90%+

Supplementary Table S3. shRNAs sequence.

Human	shCtrl	TTCTCCGAACGTGTCACGT
	shILF2	CAGGGACATTTGAAGTGCA
Mouse	shCtrl	TTCTCCGAACGTGTCACGT
	shIlf2	GCCAACATTGGAAGCTGTT
